# Preparation, Properties and Application in Electrospinning of Tremella Polysaccharide–Protein Complex

**DOI:** 10.3390/foods12081609

**Published:** 2023-04-10

**Authors:** Xiaofang Zhao, Zhiyu Wang, Yingxu Liu, Zhaolian Han, Tingting Liu, Zhiqiang Cheng

**Affiliations:** 1 School of Food Science and Engineering, Jilin Agricultural University, Changchun 130118, China; 2 Engineering Research Center of Grain Deep-Processing and High-Effeciency Utilization of Jilin Province, Changchun 130118, China; 3 School of Resources and Environment, Jilin Agricultural University, Changchun 130118, China

**Keywords:** tremella polysaccharide, protein complex, antioxidant activity, rheological property, electrostatic spinning

## Abstract

In this paper, the effects of different proteins (soybean protein isolate, wheat protein hydrolysate, tremella protein) on the activity of tremella polysaccharide under different conditions were studied. The optimal protein–polysaccharide complex was determined by grafting degree and activity screening, and the microstructure and rheological properties were studied. The results showed that when the ratio of soybean protein isolate to tremella polysaccharide was 2:1 and the solution pH was 7, the optimal complex was obtained by heating at 90 °C for 4 h, and its grafting degree and antioxidant activity were the best. Studies have shown that tremella polysaccharide and soybean protein isolate complex (TFP-SPI) solution is pseudoplastic fluids. At the same time, tremella polysaccharide (TFP) and TFP-SPI were used for electrospinning to observe its spinnability. When the ratio of PVA/TFP-SPI/PL was 8:1:1, nanofibers with uniform diameter and good morphology were obtained. This paper provides a theoretical basis for the comprehensive utilization of tremella polysaccharide and its electrospun fiber can be used as active film for food packaging.

## 1. Introduction

Tremella fuciformis is a traditional medicinal and edible fungus, which is widely used in food, medicine, and cosmetics. As its main active ingredient, tremella polysaccharide has antioxidant, anti-inflammatory, and moisturizing effects [1]. Nowadays, there are more and more studies on tremella polysaccharide, especially its antioxidant and water retention properties, which are regarded as the best natural moisturizing ingredients and one of the functional bioactive ingredients applied to food [2]. However, the poor film-forming properties of tremella polysaccharide limit its application. Studies have shown that Maillard reaction can be used for polysaccharide modification, which can change the structure of polysaccharide and improve the function of polysaccharide by reducing the carbonyl group of sugar and the amino condensation rearrangement of protein [3]. Some glycosylated products (melanoidins, heterocyclic compounds, etc.,) have certain antioxidant activity, and will also improve the antioxidant activity of the raw material itself [4]. Compared with other polysaccharide modification methods, Maillard reaction has the advantages of safety and green, so it is favored by researchers in various countries.

Protein and polysaccharide are two important biological macromolecules and important components in food [5]. Due to the interaction between protein molecules, the protein has good solubility and gelation [6]. As a natural food additive, polysaccharides have thickening, stability, emulsification, and so on [7]. After the combination of polysaccharides and proteins, other properties will also change, such as rheological properties, emulsifying properties, etc., [8].

Electrospinning is an emerging technology. The prepared nanofiber membrane has excellent properties such as high specific surface area, porosity, permeability, good biocompatibility, and degradability [9]. At the same time, electrospinning technology is a non-thermal processing technology, which has broad application prospects in food [10]. However, it is restricted by the viscoelasticity, surface tension, conductivity, and other properties of the raw materials. It is necessary to process the raw materials through certain technical means to make them suitable for electrospinning [11].

The use of electrospinning technology to prepare nano-fiber membrane of bio-based macromolecular materials as food packaging film is a development trend, is also the research focus of researchers at present, with great potential, it not only has the characteristics of biodegradability, biological activity, edible, but also can give the fiber membrane antibacterial, antioxidant, and other activities.

In this paper, the Maillard reaction treatment of tremella polysaccharide was carried out to improve its performance and enhance its spinnability. At the same time, using electrospinning technology to co-spin tremella polysaccharides and antibacterial peptides to prepare nanofiber membrane, which can be applied in food packaging, is not only a new trend of nanomaterials for food packaging, but also a new exploration of the development and utilization of edible fungi functional components, so that the comprehensive utilization of edible fungi is more meaningful.

## 2. Materials and Methods

### 2.1. Materials

Tremella polysaccharide, Tremella protein were made in laboratory (Tremella was purchased from Changchun market, Changchun, China). Soybean protein isolate (Food grade, protein mass fraction is 90%) was purchased from Henan Wanbang Chemical Technology Co., Ltd. (Shangqiu, China). Wheat protein hydrolysate (Food grade, protein mass fraction is 90%) was purchased from Shanghai Xintai Industrial Co., Ltd. (Shanghai, China). Anhydrous ethanol was purchased from Beijing Chemical Plant. O-phthalaldehyde, disodium hydrogen phosphate, sodium dihydrogen phosphate, hydrochloric acid, salicylic acid, hydrogen peroxide were purchased from Aladdin reagent company. Borax, sodium dodecyl sulfate, sulfuric acid, potassium ferricyanide, trichloroacetic acid, ferric chloride, pyrogallol, ferrous sulfate were purchased from Chemical Reagent Factory of Guoyao Group. β-Sulfoethanol, carbazole, 1,1-diphenyl-2-picrylhydrazyl (DPPH), tris (hydroxymethyl) aminomethane (Tris) were purchased from Sigma. The above reagents are analytically pure without special instructions, and the experimental water is distilled water.

### 2.2. Preparation of Tremella Polysaccharide

According to the method of Zhang et al. [12], tremella polysaccharide was extracted. First, the broken tremella was cleaned and removed, crushed by a crusher, and passed through a 120-mesh sieve. Then, with a solid–liquid ratio of 1:80, distilled water was added and soaked overnight. The obtained tremella solution was poured into a high-pressure reactor (Uwave-1000) at a temperature of 120 °C, a pressure of 1.0 MPa, and a reaction time of 40 min to obtain the extract. The extract was centrifuged to obtain the supernatant (3800 r/min, 15 min). The supernatant was deproteinized (Sevage reagent), concentrated, and precipitated with alcohol (4 times the volume of 90% ethanol solution, standing at 4 °C for 12 h).

### 2.3. Preparation of Tremella Protein

The tremella protein was extracted by alkali-soluble acid precipitation method. The tremella powder with 120 mesh sieve was added to distilled water with a solid–liquid ratio of 1:25, and the pH of the solution was adjusted to 8.0 with 0.5 mol/L NaOH for 3 h, 6000 r/min, centrifuged for 15 min, and the supernatant was taken. The pH of the solution was adjusted to 4.5 with 0.1 mol/L HCl, and the solution was precipitated for 4 h. After the precipitation was precipitated, the precipitate was centrifuged (6000 r/min, 15 min) to obtain the precipitate, and the tremella protein was freeze-dried.

According to the national standard GB5009.5-2016, the purity of precipitated dried tremella protein powder was calculated as 87.23 g/100 g.

### 2.4. Preparation of Different Protein Modified Tremella Polysaccharide Complex

Soybean protein isolate (SPI), tremella protein (TA), wheat protein hydrolysate (HWP), and tremella polysaccharide (TFP) were dissolved in distilled water at 4:1, 2:1, 1:1, 1:2, 1:4 weight ratios to obtain 0.5% (*w/v*) mixed solution. The experiment was carried out with different times, 2 h, 3 h, 4 h, 5 h, 6 h, different heating temperatures, 60 °C, 70 °C, 80 °C, 90 °C, 100 °C, and using 0.01 mol/L phosphate buffer to adjust the solution pH = 4, 6, 7, 8, 10. After reaching the reaction time, the ice bath was immediately cooled to 25 °C, and the reaction was completed. The mixed solution was centrifuged (3000 r/min, 15 min) and freeze-dried for later use.

### 2.5. Determination of Grafting Degree

According to the method of S. Tang et al. [13], 200 μL sample solution was added to 4 mL OPA solution and reacted in a water bath at 35 °C in the dark for 2 min. The absorbance of the sample at 340 nm was measured.

(1) $$\mathrm{DG}\;\left(\%\right)=\frac{A_{0}-A_{t}}{A_{0}}\times 100$$

### 2.6. Determination of Antioxidative Activity

Determination of DPPH free radical scavenging ability [14]: 0.1 mmol/L DPPH solution was prepared with anhydrous ethanol and stored in a refrigerator at 4 °C in the dark. About 0.1 mmol/L DPPH solution was mixed with 2 mL sample solution, reacted in dark at room temperature for 30 min, centrifuged, and the supernatant was taken. The absorbance A_1_ was measured at 517 nm. The above operation was repeated with distilled water instead of sample solution. The absorbance A_0_ was measured at 517 nm, and the absorbance A_2_ was measured at 517 nm with distilled water instead of DPPH solution.

(2) $$\mathrm{Clearance}\;\mathrm{rate}\;\left(\%\right)=\left(1\;-\frac{A_{1}-A_{2}}{A_{0}}\right)\times 100$$

Determination of hydroxyl radical scavenging ability [15]: 1 mL sample solution was mixed with 1 mL 9 mmol/L ferrous sulfate, 1 mL 9 mmol/L salicylic acid–ethanol solution and 1 mL 8.8 mmol/L hydrogen peroxide solution. After mixing, the reaction was carried out at 37 °C for 45 min, and the absorbance A_1_ was determined at 510 nm. The above operation was repeated with distilled water instead of hydrogen peroxide solution, and the absorbance A_2_ was determined. The above operation was repeated with distilled water instead of sample solution, and the absorbance A_0_ was determined.

(3) $$\mathrm{Clearance}\;\mathrm{rate}\;\left(\%\right)=\left(1\;-\frac{A_{1}-{\;A}_{2}}{A_{0}}\right)\times 100$$

Determination of total antioxidant capacity [16]: Take 2.5 mL phosphate buffer (pH = 6.6, 0.2 mol/L), 2.5 mL 1% potassium ferricyanide solution, fully mixed in the colorimetric tube, add 1 mL sample solution, mix well, 50 °C water bath for 20 min, add 2.5 mL 10% trichloroacetic acid, mix well, 3000 r/min, 10 min, centrifuge 2 mL supernatant, add 2 mL 1% ferric chloride solution, 2 mL distilled water, react for 30 min, measure the absorbance at 700 nm.

### 2.7. Determination of Uronic Acid Content

According to the method of Younes et al. [17], galacturonic acid standard solution (0.1 mg/mL) 0, 0.2, 0.4, 0.6, 0.8, and 1.0 mL were placed in 10 mL test tubes with stoppers, and water was added to 1 mL each. In the ice water bath, 5 mL borax-H_2_SO_4_ solution was added to each tube. After mixing, it was heated in a boiling water bath for 5 min, taken out and cooled to room temperature. Then 0.2 mL 1 mg/mL carbazole solution was added, vortexed for 30 s, and boiling water bath for 5 min. After cooling, the absorbance of each tube at 523 nm was measured to obtain the standard curve of uronic acid.

### 2.8. Analysis of Rheological Properties

The steady-state rheological properties and dynamic rheological properties of the samples were determined by rheometer (DHR-3, TA). The viscoelastic modulus of the sample was measured at 25 °C in the shear rate range of 0.1–100 s^−1^ on a 40 mm plate test bench. At the same time, the storage modulus (G′) and loss modulus (G″) of the samples in the range of 0.1–100 Hz were measured at 1 Pa pressure [18].

### 2.9. FTIR

FTIR spectroscopy (IRAffinity-1S, Shimadzu) was used to measure the infrared spectra of the samples. The 2 mg sample was mixed with 200 mg potassium bromide, and the samples were ground and mixed, and the samples were determined by FTIR spectroscopy. The wavenumber interval was 4000–400 cm^−1^, the resolution was 4 cm^−1^, and the number of scans was 32.

### 2.10. Microstructure Analysis

The morphology and microstructure of the samples were observed by scanning electron microscopy (SSX-550, Shimadzu, Kyoto, Japan). The sample (4 mm^2^) is mounted on a metal sample holder and gold plated by sputtering. Images were obtained at different magnifications using a 15 kV accelerating voltage.

The internal microstructure of the sample was observed by laser confocal microscopy (LSM980, Carl Zeiss AG, Jena Germany). The samples were stained with Nile blue and Rhodamine B, kept in dark overnight, and observed under a microscope to obtain images [19].

### 2.11. Electrostatic Spinning

PVA, ε-PL, and samples were dissolved in distilled water to obtain PVA solution, ε-PL solution, and sample solution, respectively. The PVA solution was stirred at 90 °C for 12 h using a magnetic stirrer, and the sample solution and ε-PL solution were stirred at room temperature for 12 h. Then, the PVA solution (9 wt%), the sample solution (5 wt%), and the ε-PL solution (10 wt%) were mixed in a certain proportion, and stirred at room temperature for 4 h.

#### 2.11.1. Viscosity Analysis of Electrospinning Solution

The viscosity of the electrospinning solution was measured by a rheometer (DHR-3). The viscosity of the electrospinning solution was measured in the range of 0.1–100 s^−1^ shear rate at 25 °C on a 40 mm plate test bench [20].

#### 2.11.2. Conductivity Analysis of Electrospinning Solution

Referring to the method of Krumreich et al. [21], the conductivity of electrospinning solution was measured by conductivity meter at 25 °C.

#### 2.11.3. Electrostatic Spinning

The 5 mL PVA/TFP/PL and PVA/TFP-SPI/PL spinning solutions were transferred to a syringe with a G20 spinning nozzle driven by an injection pump. The positive terminal of the high-voltage power supply is clamped to the metal tip of the syringe, and the negative terminal is connected to the drum for receiving nanofibers. In the electrospinning process, the positive voltage is 19 kV, the negative voltage is 0.56 kV, the flow rate is 0.5 mL/h, the distance between the tips is 13 cm, the ambient temperature is 30 °C, and the relative humidity is about 50%. The prepared electrospun fibers were dried and stored in a drying oven for the following SEM analysis [22,23].

#### 2.11.4. Microstructure Analysis of Electrospun Nanofiber Membrane

The experimental method is the same as Section 2.9.

### 2.12. Data Analysis

SPSS 23 statistical software was used for one-way analysis of variance, and *p* < 0.05 was used as the criterion significant difference, and Origin 9.5 software was used for plotting. All experiments were repeated three times.

## 3. Results and Analysis

### 3.1. Grafting Degree Analysis

**Figure 1:**
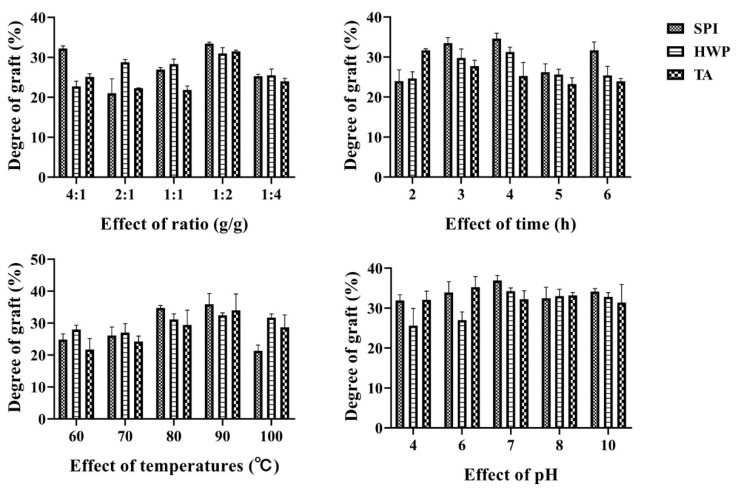
Grafting degree of complex under different treatment conditions.

The degree of grafting can be obtained by calculating the number of free amino groups during the Maillard reaction, so the degree of grafting can be used to reflect the degree of Maillard reaction [24]. Figure 1 shows the changes in grafting degree of tremella polysaccharide and three proteins under different ratios, time, temperature, and pH conditions. From the diagram, it can be seen that under different proportion conditions, the grafting degree of the three proteins and polysaccharide composites decreases first, then increases, and then decreases with the increase of the proportion. When the ratio of tremella polysaccharide to three proteins is 1:2, the grafting degree reaches the highest value. This may be because when the proportion of polysaccharides is too high, too much sugar increases the steric hindrance of the solution, which is not conducive to the reaction. When the proportion of sugar is too low, the degree of glycosylation reaction may be insufficient due to the lack of protein carbonyl donors, so the degree of grafting is low. Under the condition of different heating time, the grafting degree of soybean protein and wheat protein and tremella polysaccharide increased first and then decreased with the prolongation of time, and the grafting degree reached the maximum value at 4 h, while the tremella protein and tremella polysaccharide reached the maximum value at the initial reaction 2 h. Under different temperature conditions, the grafting degree of wheat protein and tremella polysaccharide complex did not change much, and reached the maximum at 90 °C. The grafting degree of soybean protein, tremella protein and tremella polysaccharide complex increased first and then decreased with the increase in temperature, and reached the maximum at 90 °C. This may be due to the fact that higher temperature can accelerate molecular movement, increase the probability of collision between protein and polysaccharide molecules, and contribute to the glycosylation reaction, but too high temperature may lead to protein denaturation, resulting in a decrease in grafting degree.

### 3.2. DPPH Free Radical Scavenging Ability Analysis

**Figure 2:**
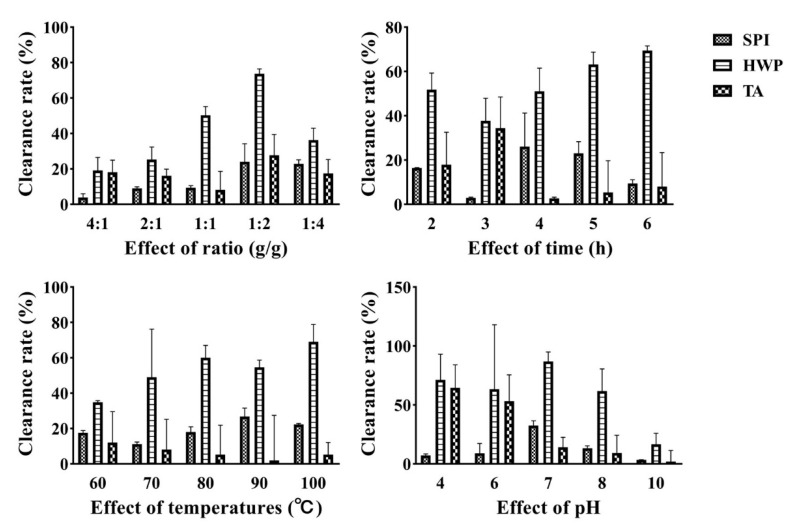
DPPH radical scavenging ability of the complex under different treatment conditions.

DPPH free radical scavenging rate test is one of the important indexes to determine the antioxidant capacity of samples, which is widely used in the study of antioxidant capacity of natural products, food, health products, and drugs [25]. Figure 2 is the DPPH free radical scavenging ability of the complex of tremella polysaccharide and three proteins under different proportions, time, temperature, and pH conditions. It can be seen from the diagram that the DPPH free radical scavenging ability of the complex of wheat protein and tremella polysaccharide is the strongest.

### 3.3. Hydroxyl Radical Scavenging Ability Analysis

**Figure 3:**
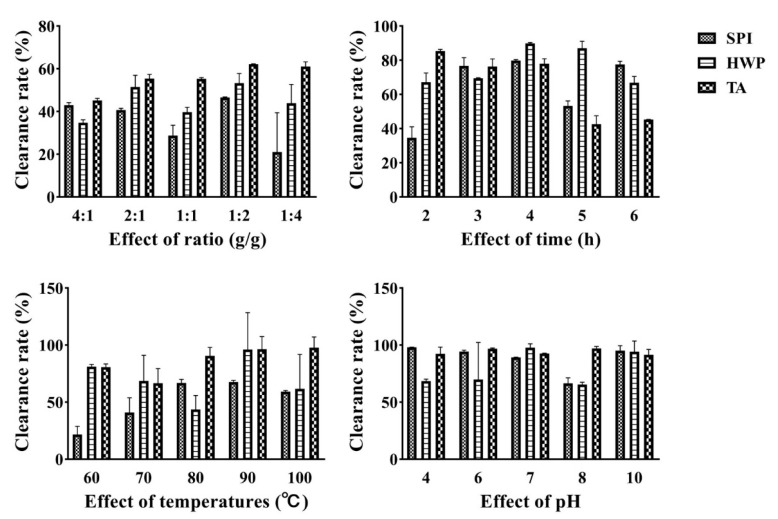
Hydroxyl radical scavenging ability of the complex under different treatment conditions.

Free radicals are a class of active chemical substances that can destroy various biological macromolecules such as DNA, proteins, and lipids in the body [26]. Figure 3 shows the comparison of hydroxyl radical scavenging ability of Tremella fuciformis polysaccharide and three proteins under different ratios, time, temperature, and pH conditions. It can be seen from the diagram that the complex of soybean protein and tremella polysaccharide has the best scavenging ability on hydroxyl radicals, which can reach more than 90%.

### 3.4. Total Antioxidant Capacity Analysis

**Figure 4:**
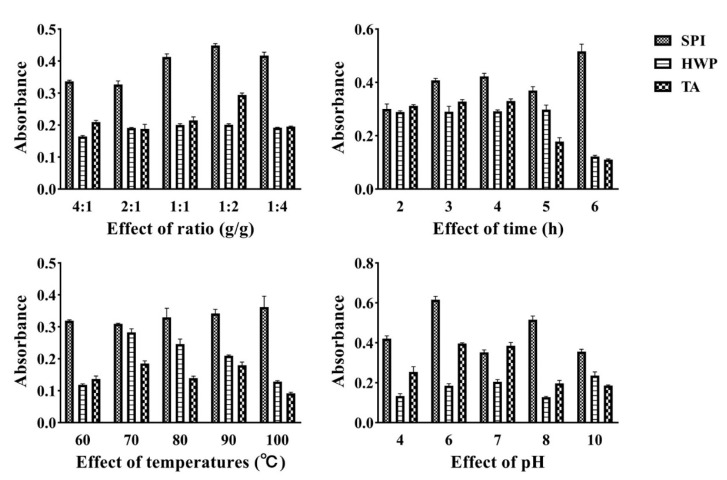
Total antioxidant capacity of the complex under different treatment conditions.

As another important index to evaluate the antioxidant properties of the material, the reducing power is usually expressed by the absorbance at 700 nm (A_700 nm_). The greater the A_700 nm_ value, the stronger the reduction ability of the material. It can be seen from Figure 4 that the total antioxidant capacity of soybean protein and tremella polysaccharide complex is much higher than that of wheat protein and tremella protein and tremella polysaccharide complex.

### 3.5. Uronic Acid Content Analysis

**Figure 5:**
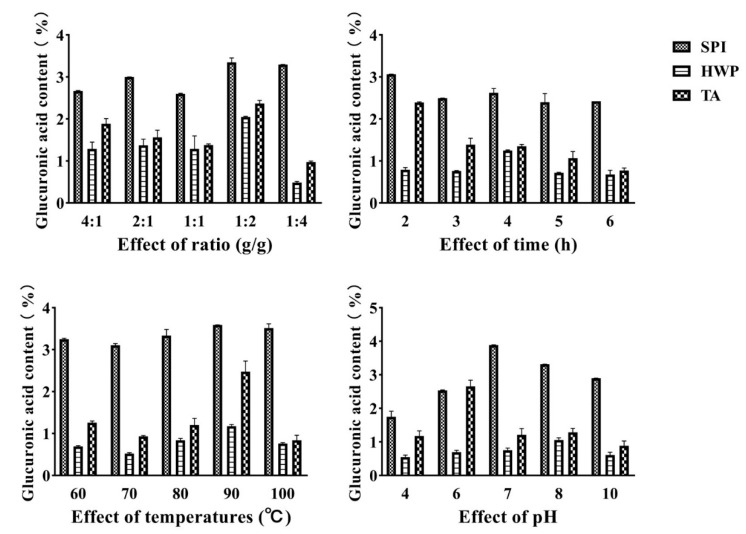
The uronic acid content of the complex under different treatment conditions.

Studies have found that the antioxidant activity of polysaccharides is related to the content of uronic acid. The carboxyl group of uronic acid is a hydrogen donor group, which can scavenge free radicals by hydrogen supply [27]. Figure 5 shows the comparison of uronic acid content of Tremella fuciformis polysaccharide and three proteins under different ratios, time, temperature, and pH conditions. From the diagram, it can be seen that the uronic acid content of soybean protein and tremella polysaccharide complex is the highest, and the total antioxidant capacity also shows this result.

In summary, according to the experimental results, the ratio of soybean protein isolate and tremella polysaccharide was 2:1, heating 4 h, 90 °C, pH = 7 was the best complex. The ratio of wheat protein hydrolysate and tremella polysaccharide was 2:1, heating 4 h, 90 °C, pH = 7 was the best condition. The ratio of tremella protein to tremella polysaccharide was 1:2, heating 2 h, 90 °C, pH = 6 was the best condition, and the subsequent test was carried out under the best conditions.

### 3.6. Rheological Properties Analysis

**Figure 6:**
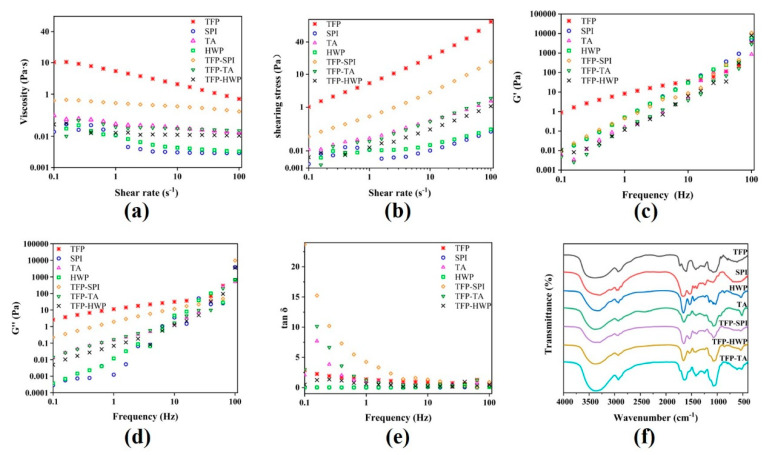
Rheological property images (**a**–**e**) and FTIR images (**f**) of different raw materials and compounds.

Viscoelastic property is an important index to reflect the internal structure and properties of polymers [28]. As shown in Figure 6, the viscosity of protein, polysaccharide, and complex decreased with the increase in shear rate, indicating that they were all pseudoplastic fluids. In the process of increasing the shear rate, the molecules are linearly arranged in the direction of shear. The main reason for pseudoplastic fluids is that proteins and polysaccharides are macromolecular colloidal particles. As the shear rate increases, the shear stress between the flow layers increases, which makes the dispersed protein–polysaccharide chain particles roll, rotate, and contract into groups, thereby reducing the hooking effect between the chain molecules and the viscosity of the protein–polysaccharide solution. The viscosity of the composite was lower than that of TFP, but the viscosity of TFP-SPI was higher than that of TFP-TA and TFP-HWP, indicating that the connection between TFP-SPI molecules was closer. Figure 6 showed the rheological curves of shear stress, apparent viscosity, and shear rate of the system, as well as the changes in storage modulus G′ and loss modulus G″ with frequency. It can be seen from the diagram that the shear stress, apparent viscosity, G′, G″ of the product after the combination of tremella polysaccharide and protein are reduced. With the increase in frequency, both G′ and G″ are increased and the dependence of complex is enhanced, indicating that the elastic modulus of complex is increased compared with that of sample. The smaller the tan δ value, the higher the degree of polymerization in the sample component, and vice versa, the lower the degree of polymerization. After the combination of tremella polysaccharide and protein, the tan δ value decreased, indicating that the interaction between tremella polysaccharide and protein occurred. The degree of polymerization increased, resulting in a strong binding force, thereby obtaining higher modulus. In particular, TFP-SPI showed the largest decrease in tan δ value, indicating high degree of polymerization between soybean protein isolate and tremella polysaccharide, which was consistent with the results of grafting degree above.

### 3.7. FTIR

FTIR is used to analyze molecular interactions between proteins and polysaccharides, such as electrostatic interactions, hydrophobic interactions, and hydrogen bonds. The infrared spectra of tremella polysaccharide and soy protein isolate, wheat protein hydrolysate and tremella protein complex are shown in Figure 6. It can be seen from Figure 6 that tremella polysaccharide has sugar characteristic peaks, such as the stretching vibration region of C-H at 3200–2800 cm^−1^, the C-H variable angle vibration region at 1400–1200 cm^−1^, and the stretching vibration absorption peaks formed by C-O-H and C-O-C of pyran ring at 1200–1000 cm^−1^ [29]. SPI, TA, HWP are the stretching vibration of -OH at 3500–3200 cm^−1^; the amide I band (stretching vibration of C=O) and amide II band (bending vibration of -NH) exist at 1700–1600 cm^−1^ and 1300–1200 cm^−1^, and the absorption peak at 520 cm^−1^ is the -SH stretching vibration peak [30]. The FTIR profiles of the complex and tremella polysaccharide and corresponding proteins showed no appearance or disappearance of specific absorption peaks, which was consistent with the results of previous studies [31]. Compared with the absorption peaks of polysaccharide and protein at 3200–3300 cm^−1^, the absorption intensity of the complex increased and shifted towards the direction of low wave number, indicating that hydrogen bond interaction was enhanced and intermolecular interaction was increased, and macroscopically, the grafting degree and polymerization degree of the complex increased. This is consistent with the previous results of graft degree and rheological properties.

### 3.8. Microstructure Analysis

**Figure 7:**
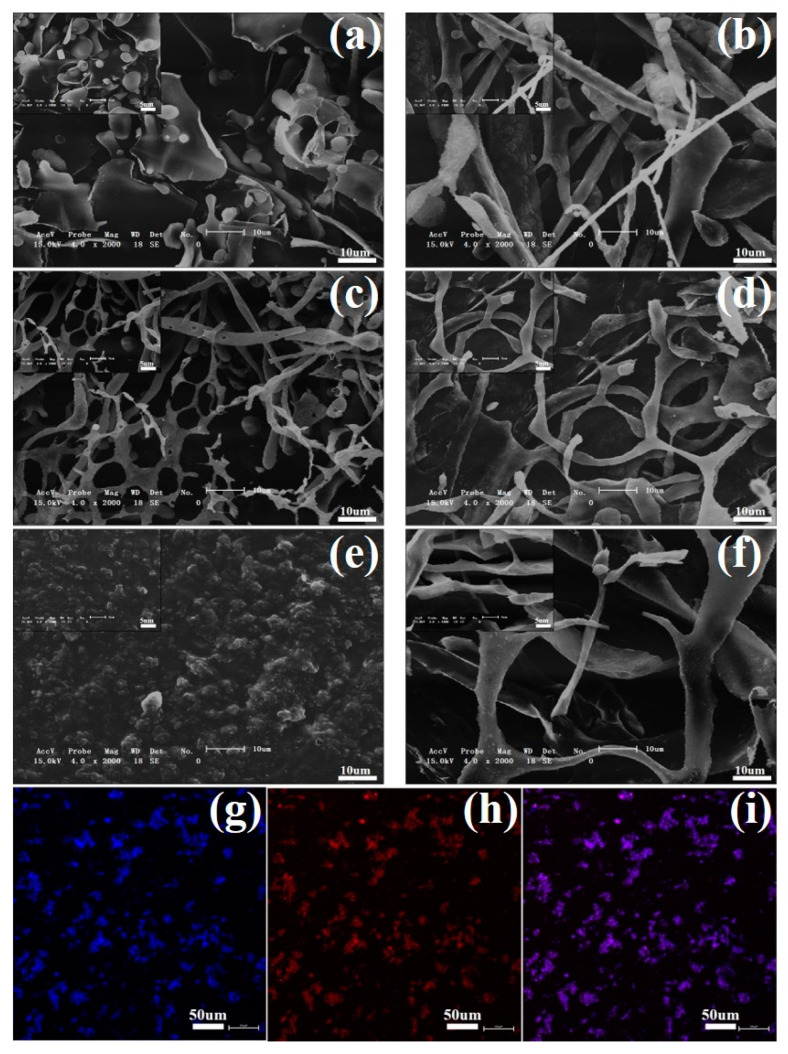
SEM images (**a**–**f**) and confocal laser images (**g**–**i**) of different raw materials and compounds. (**a**). SPI; (**b**). TFP-SPI; (**c**). HWP; (**d**). TFP-HWP; (**e**). TA; **f**. TFP-TA; (**g**). TFP staining image; (**h**). SPI staining image; (**i**). TFP-SPI solution staining image.

Figure 7 is the scanning electron microscope comparison diagram of protein and complex. It can be seen from Figure 7 that the complex of protein and polysaccharide presents a three-dimensional network structure with different numbers of holes that penetrate and cross-link each other. The complex of tremella polysaccharide and soybean protein had the least voids and the most compact and uniform structure, indicating that the interaction was the strongest [31], which was consistent with the above experimental results. Studies have shown that the interaction between polysaccharides and proteins is mainly based on hydrogen bonds, which is mainly due to the strong hydrophilicity of tremella polysaccharides, while the protein and polysaccharide molecular chains contain a large number of hydroxyl, amino, carbonyl, and other groups, which are prone to hydrogen bond interactions.

### 3.9. UV Absorbance Analysis

**Figure 8:**
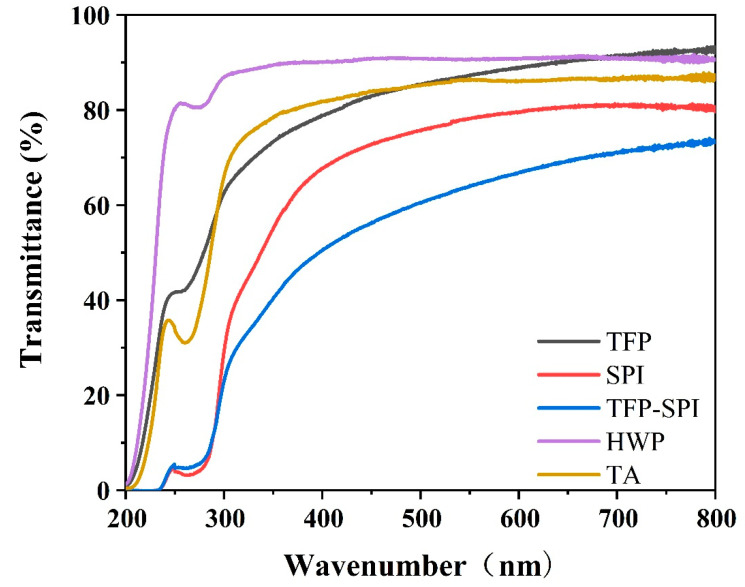
Ultraviolet spectroscopic spectra of different substances.

In order to verify the microscopic conformation of soybean protein and Tremella fuciformis polysaccharide, the complex was scanned by ultraviolet absorption spectroscopy. The tyrosine and tryptophan residues that constitute the protein have ultraviolet absorption properties. When the microenvironment of the protein molecule changes, the amino acid residue conformation changes, which in turn changes the ultraviolet absorption spectrum of the group [32]. The interaction between protein and polysaccharide will cause a shift in absorption peak and a change i absorbance. These changes are highly sensitive to environmental changes near 280 nm. As shown in Figure 8, tremella polysaccharide has no ultraviolet absorption at 280 nm, and soybean protein and tremella polysaccharide complex has an absorption peak at 280 nm, indicating that tremella polysaccharide interacts with soybean protein.

The following electrospinning was performed using the best complex of tremella polysaccharide and soy protein isolate.

### 3.10. Viscosity Analysis of Electrospinning Solution

**Figure 9:**
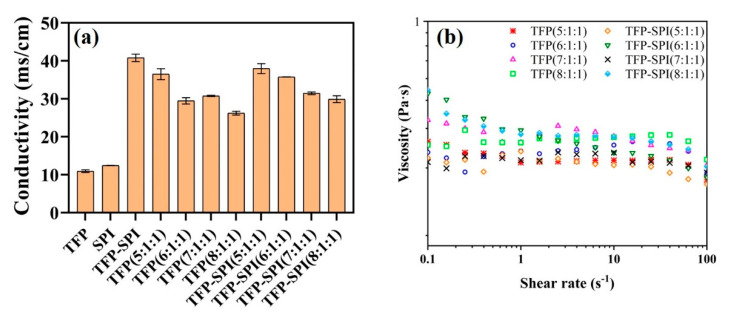
Electrical conductivity (**a**) and viscosity (**b**) of the electrospinning solution.

The conductivity and apparent viscosity of polymer solution are of great significance for the influence of fiber morphology and diameter. The study found that when the viscosity of the solution is very low, continuous fibers cannot be obtained, and when the viscosity of the solution is too high, the solution is difficult to eject from the syringe tip and cannot be electrospun [33]. It can be seen from Figure 9 that the viscosity of the electrospinning solution gradually decreases with the increase in shear rate, showing a shear thinning behavior, which is a pseudoplastic fluid, indicating that the molecules in the blend solution are entangled with each other.

### 3.11. Conductivity Analysis of Electrospinning Solution

Figure 9 shows the conductivity of the electrospinning solution. It can be seen from Figure 9 that as the concentration of the polymer increases, the conductivity decreases. The electrospinning solution with the addition of the composite has a higher conductivity than the electrospinning solution with the addition of tremella polysaccharide. Appropriate conductivity helps to form nanofibers with good morphology and uniform diameter.

### 3.12. Microstructure Analysis of Electrospun Nanofiber Membrane

**Figure 10:**
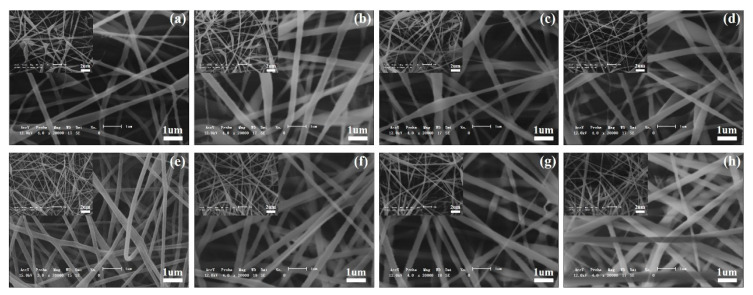
SEM images of nanofibers under different condition. (**a**). PVA/TFP/PL(5:1:1); (**b**). PVA/TFP/PL(6:1:1); (**c**). PVA/TFP/PL(7:1:1); (**d**). PVA/TFP/PL(8:1:1); (**e**). PVA/TFP-SPI/PL(5:1:1); (**f**). PVA/TFP-SPI/PL(6:1:1); (**g**). PVA/TFP-SPI/PL(7:1:1); (**h**). PVA/TFP-SPI/PL(8:1:1).

Studies have shown that for the spinning solution, too high viscosity and too low conductivity will hinder the stretching of the fiber during the jetting process. The resulting fiber has a large and uneven diameter, and even causes the droplets to accumulate at the jetting pinhole of the syringe and cannot be electrospun [11]. Figure 10 is the scanning electron microscope images of electrospun nanofibers with different proportions of tremella polysaccharide and different proportions of composite. It can be seen from the figure that as the proportion of the polymer increases, the fiber becomes more and more uniform, and the beads decrease. When the ratio of PVA/TFP-SPI/PL reaches 8:1:1, the fiber diameter is uniform and no beads are produced.

## 4. Conclusions

In this paper, the complexes of protein and polysaccharide were successfully prepared, and the complexes with the best grafting degree and antioxidant activity were screened, that is, the ratio of soybean protein to tremella polysaccharide was 2:1, heating 4 h, 90 °C, pH = 7. The UV absorption spectra showed that the composite had an absorption peak at 280 nm, indicating the interaction between TFP and SPI. FTIR showed that the absorption peak of the complex widened and shifted at 3200–3300 cm^−1^, indicating that there was hydrogen bond interaction between polysaccharide and protein. Scanning electron microscopy (SEM) images showed that the complex presented a three-dimensional network structure of interpenetration and crosslinking. The rheological properties of the composite were tested, and it was inferred that the composite solution was pseudoplastic fluid. At the same time, uniform bead-free nanofibers were prepared by electrospinning technology, which could be used as active films for food packaging in the future.

## Data Availability

Data is contained within the article.

## References

[1] Ju A., Song K.B. (2020). Active biodegradable films based on water soluble polysaccharides from white jelly mushroom (*Tremella fuciformis*) containing roasted peanut skin extract. LWT.

[2] Tang R., Lu Y., Hou C., Peng J., Wang W., Guo X. (2020). Co-Supplementation of Flos Sophorae Extract with Tremella fuciformis Polysaccharides Im-proves Physicochemical, Textural, Rheological, and Antioxidant Properties of Low-Fat Yogurts. J. Food Qual..

[3] Li Q., Li X., Ren Z., Wang R., Zhang Y., Li J., Ma F., Liu X. (2021). Physicochemical properties and antioxidant activity of Maillard reaction products derived from Dioscorea opposita polysaccharides. LWT.

[4] Luo Y., Tu Y., Ren F., Zhang H. (2022). Characterization and functional properties of Maillard reaction products of β-lactoglobulin and polydextrose. Food Chem..

[5] Ke C., Li L. (2023). Influence mechanism of polysaccharides induced Maillard reaction on plant proteins structure and functional properties: A review. Carbohydr. Polym..

[6] Oliver C.M., Melton L.D., Stanley R.A. (2006). Creating Proteins with Novel Functionality via the Maillard Reaction: A Review. Crit. Rev. Food Sci. Nutr..

[7] Kan X., Chen G., Zhou W., Zeng X. (2021). Application of protein-polysaccharide Maillard conjugates as emulsifiers: Source, preparation and functional properties. Food Res. Int..

[8] Han M.-M., Yi Y., Wang H.-X., Huang F. (2017). Investigation of the Maillard Reaction between Polysaccharides and Proteins from Longan Pulp and the Improvement in Activities. Molecules.

[9] Xie Q., Liu G., Zhang Y. (2022). Edible films/coatings containing bioactive ingredients with micro/nano encapsulation: A compre-hensive review of their fabrications, formulas, multifunctionality and applications in food packaging. Crit. Rev. Food Sci. Nutr..

[10] Li J., Zhang F., Zhong Y., Zhao Y., Gao P., Tian F., Zhang X., Zhou R., Cullen P.J. (2022). Emerging Food Packaging Applications of Cellulose Nanocomposites: A Review. Polymers.

[11] Majidi S., Movaffagh J., Kamali H., Shahroodi A., Tafaghodi M., Salarbashi D. (2022). Development and characterization of sumatriptan-loaded soy bean polysaccharide nanofiber using electrospinning technique. J. Drug Deliv. Sci. Technol..

[12] Zhang S., Yang J., Zhao W., Liu H., Liu T. (2020). Optimization, Structure Identification and Antioxidant Activity Analysis of Polysaccha-rides from Tremella Fuciformis by Pressurized Hot Water Extraction Method. J. Jilin Agric. Univ..

[13] Tang S., Jiang Y., Tang T., Du H., Tu Y., Xu M. (2022). Effects of Grafting Degree on the Physicochemical Properties of Egg White Protein-Sodium Carboxymethylcellulose Conjugates and Their Aerogels. Appl. Sci..

[14] Zhang C., Guo X., Guo R., Zhu L., Qiu X., Yu X., Chai J., Gu C., Feng Z. (2023). Insights into the effects of extractable phenolic compounds and Maillard reaction products on the antioxidant activity of roasted wheat flours with different maturities. Food Chem. X.

[15] Özcan M.M. (2022). The Effect of Spice Powders on Bioactive Compounds, Antioxidant Activity, Phenolic Components, Fatty Acids, Mineral Contents and Sensory Properties of “Keşkek”, Which Is a Traditional Food. Foods.

[16] Gao W., Zhang N., Li S., Li S., Zhu S., Cong X., Cheng S., Barba F.J., Zhu Z. (2022). Polysaccharides in Selenium-Enriched Tea: Extraction Performance under Innovative Technologies and Antioxidant Activities. Foods.

[17] Younes A., Karboune S., Liu L., Andreani E.S., Dahman S. (2023). Extraction and Characterization of Cocoa Bean Shell Cell Wall Polysaccharides. Polymers.

[18] Li Z., Zhou B., Zheng T., Zhao C., Gao Y., Wu W., Fan Y., Wang X., Qiu M., Fan J. (2023). Structural Characteristics, Rheological Properties, and Antioxidant and Anti-Glycosylation Activities of Pectin Polysaccharides from Arabica Coffee Husks. Foods.

[19] Tu J., Adhikari B., Brennan M.A., Cheng P., Bai W., Brennan C.S. (2023). Interactions between sorghum starch and mushroom polysaccharides and their effects on starch gelatinization and digestion. Food Hydrocoll..

[20] Ghorani B., Emadzadeh B., Rezaeinia H., Russell S. (2020). Improvements in gelatin cold water solubility after electrospinning and associated physicochemical, functional and rheological properties. Food Hydrocoll..

[21] Krumreich F.D., Prietsch L.P., Antunes M.D., Jansen-Alves C., Mendonça C.R.B., Borges C.D., Zavareze E.d.R., Zambiazi R.C. (2019). Avocado Oil Incorporated in Ultrafine Zein Fibers by Electrospinning. Food Biophys..

[22] Liu F., Liu Y., Sun Z., Wang D., Wu H., Du L., Wang D. (2020). Preparation and antibacterial properties of ε-polylysine-containing gelatin/chitosan nanofiber films. Int. J. Biol. Macromol..

[23] Shao P., Niu B., Chen H., Sun P. (2018). Fabrication and characterization of tea polyphenols loaded pullulan-CMC electrospun nanofiber for fruit preservation. Int. J. Biol. Macromol..

[24] Ping-Ping W., Wen-Duo W., Chun C., Xiong F., Rui-Hai L. (2020). Effect of Fructus Mori. bioactive polysaccharide conjugation on improving functional and antioxidant activity of whey protein. Int. J. Biol. Macromol..

[25] Song L., Liu S., Zhang L., Pan L., Xu L. (2023). Polysaccharides from Nitraria retusa Fruit: Extraction, Purification, Structural Characterization, and Antioxidant Activities. Molecules.

[26] Yang Z., Zeng Y., Hu Y., Zhou T., Li J., He L., Zhang W., Zeng X., Fan J. (2023). Comparison of chemical property and in vitro digestion behavior of polysaccharides from Auricularia polytricha mycelium and fruit body. Food Chem. X.

[27] Peng Z., Tian S., Li H., Zhu L., Zhao Z., Zheng G., Wen Q., Tian H., Yang D. (2023). Extraction, characterization, and antioxidant properties of cell wall polysaccharides from the pericarp of Citrus Reticulata cv. Chachiensis. Food Hydrocoll..

[28] Zhang J., Wang G., Liang Q., Cai W., Zhang Q. (2019). Rheological and microstructural properties of gelatin B/tara gum hydrogels: Effect of protein/polysaccharide ratio, pH and salt addition. LWT.

[29] Yang F., Du Q., Miao T., Zhang X., Xu W., Jia D. (2022). Interaction between potato starch and Tremella fuciformis polysaccharide. Food Hydro-Colloids.

[30] Zhao H., Zhao R., Liu X., Zhang L., Liu Q., Liu W., Wu T., Hu H. (2023). Effect of high intensity ultrasonic treatment on structural, rheological, and gelling properties of potato protein isolate and its co-gelation properties with egg white protein. J. Food Sci..

[31] Feng J., Tian H., Chen X., Cai X., Shi X., Wang S. (2023). Interaction between fish gelatin and tremella polysaccharides from aqueous solutions to complex coacervates: Structure and rheological properties. Food Hydrocoll..

[32] Bishnoi S., Trifol J., Moriana R., Mendes A.C. (2022). Adjustable polysaccharides-proteins films made of aqueous wheat proteins and alginate solutions. Food Chem..

[33] Vargas-Campos L., Figueroa-Cárdenas J.D.D., Tochihuitl-Vázquez D., Ramírez-Bon R., Yáñez-Limón J.M., Pérez-Robles J.F. (2023). Study of the dextrose equivalent of maltodextrins in electrospinning using an ethanol/water mixture as the electrospinning solvent. Food Hydrocoll..

